# Multi-Crosslinked Strong and Elastic Bioglass/Chitosan-Cysteine Hydrogels with Controlled Quercetin Delivery for Bone Tissue Engineering

**DOI:** 10.3390/pharmaceutics14102048

**Published:** 2022-09-26

**Authors:** Qing Min, Ronghua Tan, Yuchen Zhang, Congcong Wang, Ying Wan, Jing Li

**Affiliations:** 1 School of Pharmacy, Hubei University of Science and Technology, Xianning 437100, China; 2 College of Life Science and Technology, Huazhong University of Science and Technology, Wuhan 430074, China; 3 School of Medicine, Huzhou University, Huzhou 313000, China

**Keywords:** multi-crosslinked hydrogel, chitosan-cysteine conjugate, amino-functionalized bioglass nanoparticles, quercetin, strength and elasticity, controllable and sustainable release

## Abstract

Chitosan-cysteine (CH-CY) conjugate with an optimal content of thiol groups was synthesized and combined with amino-functionalized mesoporous bioglass (ABG) nanoparticles (NPs) with radially-porous architecture to build multi-crosslinked ABG/CH-CY composite hydrogels. Besides the network formed by self-crosslinking of thiol groups in CY-derived side chains, difunctionalized PEG (DF-P) crosslinkers with varying lengths of PEG segments were used to crosslink amino groups on CH-CY or ABG NPs to form other networks in the composite gels. Quercetin (Que) was loaded into ABG NPs before these NPs were incorporated into the hydrogel, intending to achieve sustainable and controllable Que release from so-built ABG/CH-CY gels. The lengths of PEG segments in DF-P were found to impose remarkable impacts on the strength or elasticity of multi-crosslinked ABG/CH-CY hydrogels. Some ABG/CH-CY hydrogels had their elastic modulus of around 8.2 kPa or higher along with yielding strains higher than 70%, specifying their mechanically strong and elastic characteristics. In addition, these gels showed the ability to release Que and Si or Ca ions in controllable ways for various durations. The optimally achieved ABG/CH-CY hydrogels were injectable and also able to support the growth of seeded MC3T3-E1 cells as well as the specific matrix deposition. The obtained results suggest that these ABG/CH-CY gels have promising potential for bone repair and regeneration.

## 1. Introduction

Injuries to bone tissues often occur in a variety of situations, primarily including bone fractures, trauma, infections, tumor excision, congenital malformations, or skeletal diseases; severe bone damage can lead to lifelong disability in many patients [1]. Bone autografting is generally acknowledged as an ideal approach for bone repair but it is hindered on account of the limited donor availability and possible donor-site morbidity [2]. As for allografting, despite its availability, allografting may expose patients to several potential risks such as immunogenicity, disease transmission, and a high incidence of non-union healing [3,4].

In search of alternative therapies for bone injuries, biomaterial-based tissue engineering technology has emerged as a promising option for clinical bone repair and regeneration [5,6]. Among various forms of biomaterials, injectable polymer hydrogels with biocompatibility and biodegradability have attracted a lot of interest in bone tissue engineering because they can fill irregular bone defects with discretional shapes and form into self-supporting objects in situ during tunable periods via a minimally invasive injection procedure [7,8]. Besides these, they behave similar to liquids before injection and can in situ transform into solid-like fillers with interconnected porous structure, high water retention, and good permeability after injection, making them particularly conducive to delivering cells, drugs, and bioactive molecules as well as easy transport of nutrients and metabolites [7,8,9]. Nowadays, various kinds of natural polymers, commonly including collagen, gelatin, silk fibroin, chitosan (CH), dextran, alginate, and hyaluronic acid, have been extensively investigated for hydrogel applications as they are gelable while showing adequate biocompatibility and easy biodegradation [8,10,11], and meanwhile, these natural polymer-based hydrogels are found to have much better biological performance in many cases than those synthetically sourced polymer hydrogels [10,11].

Of mentioned natural polymers, CH has been intensively investigated as an injectable scaffold material for a wide range of biomedical applications due to its well-demonstrated advantages, such as non-toxicity, non-antigenicity, anti-microbial nature, bio-adherence, and cell affinity [12,13]. In particular, CH molecules have their chemical structure closely similar to glycosaminoglycans (GAGs), a kind of tissue component existing in many types of extracellular matrix (ECM). Due to these distinctive biological properties of CH, extensive research has gone into the development of CH-based hydrogels so far [13,14], and many of them have already been utilized for the repair and regeneration of injured skin, nerve, cartilage, and bone [11,12,13,14,15]. Despite the wide-ranging usability, the hydrogels based on innate CH often show low strength, poor elasticity, and fast in vivo degradation, which limits their application in repairing certain tissue injuries where the establishment of a sufficiently strong and elastic microenvironment for housing the seeded or migrated cells is specifically required.

Many studies have revealed that multi-component hydrogels with dual or multi-network structures could be substantially enhanced in their mechanical performance and degradation tolerance through chain entanglement, intermolecular interactions, and mutual network restriction provided that the right components for the gel construction are selected while employing suitable physical or chemical crosslinking techniques [16,17,18]. Hence, it should be feasible to combine CH with other ingredients or utilize modified CH rather than the innate CH to build certain strong and elastic CH-based hydrogels. Chitosan-cysteine (CH-CY) conjugate is a kind of CH-derivatives and it contains both amino and thiol groups, and therefore, CH-CY itself can be processed into dual network hydrogels by crosslinking its amino or thiol groups [19,20,21]. Nevertheless, the gels built by crosslinking only a single CH-CY component have mechanically weak attributes with high swelling [19,22].

For the hydrogel intended for use in bone repair, apart from endowing it with excellent mechanical properties, compounding certain pro-osteogenic ingredients is one of the practical strategies for promoting its bone repair ability. To date, bioglass (BG) nanoparticles (NPs) have been commonly used together with hydrogels for bone repair because of their several meritorious properties: (i) they can transform into hydroxyapatite-like substances under the action of physiological fluids and firmly bond to the bone tissue at the defect [23]; (ii) their certain dissolution products have bioactivity in stimulating osteogenesis and angiogenesis [23,24,25,26]; and (iii) they could mechanically enhance hydrogels as long as they are effectively cross-linked with the polymer networks in the gels [23,27]. Bioactive glasses are usually composed of calcium-containing silicates, but many other kinds of bioactive glasses with different element doping have also been developed. Typical doping elements include phosphate, silver, copper, zinc, magnesium, selenium, strontium, and boron [28]. These various doped bioactive glasses are being explored for varieties of biomedical applications [28,29,30].

Owing to the presence of amino and thiol groups in CH-CY, it can thus be envisaged that thiol- or amino-functionalized BG NPs would be a suitable ingredient to combine with CH-CY conjugate for building multi-crosslinked BG/CH-CY hydrogels with improved mechanical properties and additional functions as well. By employment of mesoporous BG NPs for constructing BG/CH-CY hydrogel, it becomes possible to use the resulting gels as a vehicle for delivering drugs or bioactive molecules since mesoporous BG NPs can serve as a reservoir for loading the required therapeutic agents. Quercetin (Que) is a kind of naturally sourced flavonoid compound and it shows a wide spectrum of biological activities [31,32], including antioxidation, anti-inflammation, anti-hypertension, anti-carcinogenicity, immunomodulatory actions, and so on. Que has also been used for bone repair due to its functions for promoting osteogenesis, anti-oxidation, anti-inflammation, and so on [33,34,35,36,37,38].

**Scheme 1:**
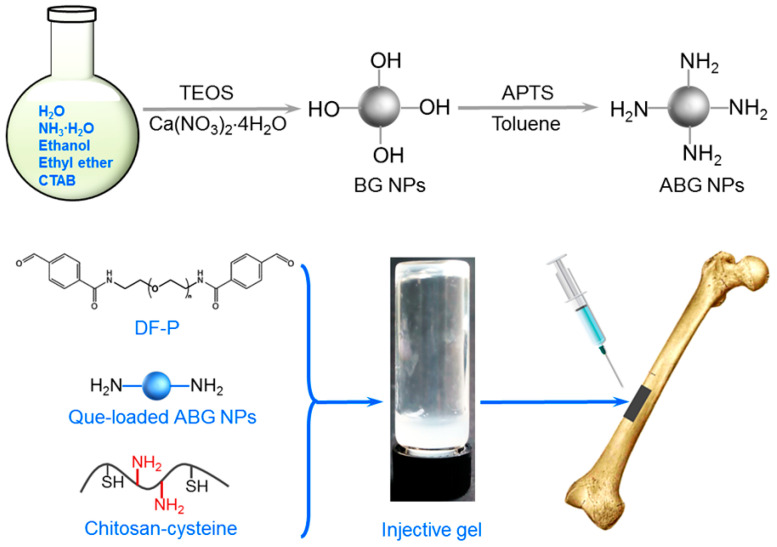
Schematic illustrations for the synthesis of mesoporous BG and ABG bioglass NPs, preparation of composite hydrogel, and the intended use in bone repair.

In this context, it would be rational to develop a new type of composite hydrogel with strong and elastic nature while having the ability to sustainably deliver bioactive ions and Que to the defect site for the intended use in bone repair. Accordingly, Que was first loaded into amino-functionalized mesoporous BG (ABG) NPs, and the resulting ABG NPs were then combined with CH-CY to construct multi-crosslinked composite hydrogels using difunctionalized polyethylene glycol (dibenzaldehyde-terminated PEG, DF-P) with various lengths of PEG segment as a crosslinker. In these composite hydrogels, the thiol groups in CH-CY can be self-crosslinked to form a network, and the amino groups in CH-CY or ABG NPs can be cross-linked by the terminal aldehyde groups in DF-P molecules to build more networks inside the gels. Schematic illustrations for synthesizing BG and ABG NPs and the composition of resulting composite gel as well as potential gel application in bone repair are shown in Scheme 1. Some optimally constructed gels were found to have mechanically strong and elastic characteristics while showing a well-defined ability to administer the release of Si or Ca ions and Que. They were also able to support the growth of seeded MC3T3-E1 cells and matrix deposition. The results suggest that these composite hydrogels have the potential to function as an injectable material for bone tissue engineering.

## 2. Materials and Methods

### 2.1. Materials

CH, Que, L-cysteine (CY), 3-(aminopropyl)triethoxysilane (APTES), tetraethyl orthosilicate (TEOS), *N*-hydroxysuccinimide (NHS), cetyltrimethylammonium bromide (CTAB), and 1-ethyl-3-(3-dimethylaminopropyl carbodiimide) hydrochloride (EDC) were supplied by Aladdin Inc. (Shanghai, China). The purchased CH was further treated in a 50 wt% NaOH aqueous solution to increase its degree of deacetylation (DDA) using the method described in our previous study [39], and the DDA and viscosity-average molecular weight of the treated CH were measured to be around 94.8% and 4.9 × 10^5^, respectively. DF-P (benzaldehyde-PEG-benzaldehyde) crosslinkers with various lengths of PEG segment were purchased from Ponsure Biotechnology, Ltd. (Shanghai, China), and they were referred to as DF-P1000, DF-P3400, and DF-P5000, respectively, where the number following the DF-P indicates the Mn of PEG segments. Other chemicals were of analytical grade and purchased from Sinopharm (Shanghai, China).

Mesoporous BG NPs were synthesized using methods similar to that described in our previous study [27]. The amino-functionalization of BG NPs was conducted by reacting BG NPs with APTS in dry toluene. In a typical process, BG NPs (30 mg) were suspended in 20 mL of dry toluene with ultrasonic treatment for 30 min, and 0.30 mL of APTS was then added to this suspension with stirring at 80 °C for 6 h. Amino-functionalized BG (ABG) NPs were retrieved by centrifugation, washed with dry toluene and methanol, air-dried for 24 h, and dried again at 45 °C under reduced pressure for 24 h. The content of amino groups in ABG NPs was determined with a ninhydrin assay [40].

Two kinds of Que solutions (100 or 200 mg/mL) in anhydrous ethanol were first produced and they were then used for the preparation of Que-loaded NPs. In brief, 2 mL of either Que solution was introduced into an EP tube that contains a prescribed amount of ABG NPs, and the mixture was sonicated for 10 min to facilitate dispersion of NPs. Afterward, the tube was shaken on an orbital table for 24 h at 37 °C and 60 rpm under light-shielding conditions. The Que-loaded ABG NPs were retrieved by centrifugation at 8000 rpm for 10 min, washed with 30% ethanol and PBS, freeze-dried, and dried again in an oven at 60 °C under reduced pressure for 24 h. By changing the mass ratio of Que to ABG NPs, four kinds of Que-loaded ABG NPs were prepared, and they were entitled ABG-Q1, ABG-Q2, ABG-Q3, and ABG-Q4, respectively. The amount of Que loaded was determined by measuring the difference of Que concentrations in the loading medium before and after soaking ABG NPs by using spectrophotometric measurement [34,41]. Drug load (DL) was calculated using the following formula:

DL = [M_0_/M] × 100% (1)

where M_0_ is the mass of Que loaded inside NPs, and M denotes the mass of NPs.

### 2.2. Synthesis of Chitosan-Cysteine Conjugate

CH-CY conjugates were synthesized by grafting CY onto the C-2 sites of the CH backbone. Typically, 0.726 g CY (-COOH: ca. 6.0 mmol) was dissolved in *N*,*N*-dimethylformamide (10 mL), followed by the addition of 2.3 g of EDC (ca. 12.0 mmol) and 1.381 g of NHS (ca. 12.0 mmol). The resulting solution was activated for 6 h at room temperature and then introduced into a CH solution (514 mg of CH in 40 mL 0.1 M HCl; -NH_2_: ca. 3.0 mmol) with stirring. The pH value of the reaction system was adjusted to around 5 using a 1.0 M NaOH solution. After reaction for 24 h at room temperature, the synthesized CH-CY conjugate was dialyzed against 5 mM HCl solution for 12 h, and then against 5 mM HCl solution containing 1% NaCl for an additional 12 h. It was further dialyzed against 1 mM HCl solution for 12 h, followed by freeze-drying. The reactions and dialysis were conducted in the dark and the dialysis temperature was maintained at 4 °C. Different CH-CY conjugates with varied substitution degrees of thiol groups were synthesized by changing the molar ratio of carboxyl groups in CY to amino groups in CH. The content of thiol groups in CH-CY conjugates was measured by Ellman’s method [42], and the substitution degree of thiol groups in CH-CY conjugates was determined by comparing the peak area between methylene protons on -CH_2_SH and C-2 protons of glucosamine units in CH.

### 2.3. Characterization

Fourier transform infrared (FTIR) spectra of samples were detected using a spectrometer (Vertex70, Bruker, Ettlingen, Germany) in the transmission mode. CH-CY conjugates were dissolved in D_2_O to reach a concentration of 10 mg/mL and their ^1^H NMR spectra were recorded on an NMR spectrometer (Avance600, Bruker, Rheinstetten, Germany). A transmission electron microscope (TEM, Tecnai, FEI, Hillsboro, OR, USA) was employed to observe the morphology, size, and dispersion of NPs. A dynamic light scattering (DLS) instrument (Nano-ZS90, Malvern, Worcestershire, UK) was used to detect the hydrodynamic size and zeta (ζ) potential of NPs. For the measurements of isotherms and pore-size distributions, NPs were first dried in a vacuum oven at 100 °C for 12 h before loading into the sample chamber of the surface area and pore size analyzer (ASAP2020 Plus, Micromeritics, Norcross, GA, USA). After being degassed at 120 °C for 6 h, the volume of nitrogen adsorption-desorption was measured at different pressures. The specific surface areas of NPs were determined using the BET method, and the corresponding pore size was calculated with the BJH method.

### 2.4. Preparation of Hydrogels

CH-CY hydrogels without incorporation of NPs were prepared as follows. Briefly, the selected CH-CY was dissolved in deionized water to prepare CH-CY solutions with various concentrations. Aliquots (1 mL) of CH-CY solutions were introduced into glass vials placed in an ice/water bath and each solution was mixed with a given amount of DF-P1000 to prepare a series of mixtures. The pH values of the mixtures were adjusted to about 7 using a 5% NaHCO_3_ solution with stirring for 5 min, and the vials were then moved to an incubator for gelling the mixtures at 37 °C.

In the case of ABG/CH-CY composite hydrogel preparation, Que-loaded ABG NPs were added to a CH-CY solution (2.5 *w*/*v*%), followed by the addition of DF-P with various lengths of PEG segment as a crosslinker. So prepared composite solutions were introduced into different vials in an ice/water bath with stirring and their pH values were also adjusted to around 7 using a 5% NaHCO_3_ solution. After that, the vials were incubated at 37 °C for gel formation.

### 2.5. Rheological Measurements

Rheological measurements were conducted on a rheometer (Kinexus Pro KNX2100, Southborough, MA, USA). Spectra for elastic modulus (G′) or viscous modulus (G″) versus frequency were detected in a range between 0.1 and 100 Hz at 37 °C with a constant strain of 1%. Strain sweep spectra for G′ and G″ of gels were detected by setting the temperature at 37 °C and frequency at 1 Hz. Shear viscosity sweeps for gels were conducted in a shear rate range between 0.1 and 200 s^−1^ at 25 °C using liquid samples.

### 2.6. Release of Ions and Que

The cylindrical ABG/CH-CY gel samples (0.5 mL, diameter: 10 mm) were first produced. These gels were respectively introduced into different vials that were filled with 5 mL of PBS, and the vials were vortexed on the shaking table at 37 °C and 60 rpm. At each predetermined sampling point, 1 mL of supernatant was withdrawn and an equal volume of fresh release buffer was replenished. The collected supernatants were diluted 20, 40, 80, and 160 times according to their concentration differences so that the concentrations of the dilutions were within the linear range of the respective standard curves of different ions. These dilutions were then assayed using inductively coupled plasma atomic emission spectrometry (ICP-AES, SPECTRO CIROS-CCP, Kleve, Germany) to measure the released amount of ions. Standard curves for Si or Ca ions were established using standard solutions containing silicon or calcium compounds. In the case of Que release measurements, the released amount of Que from gels was detected using spectrophotometric methods [34,41]. In brief, a standard curve for Que was first generated using Que solutions in ethanol with a concentration gradient series (19.2, 23.0, 32.9, 38.3, 46.0, and 57.5 μg/mL) and absorbance of these Que solutions was detected at 256 nm. At each prescribed time point, an aliquot of release medium was taken with replenishment of an equal amount of fresh release buffer. The aliquot was diluted to the desired concentration so that its absorbance was registered within the linear range of the standard curve. The concentration of the released Que in the aliquot was spectrophotometrically determined.

### 2.7. Cell Culture

MC3T3-E1 cells (Type Culture Collection of the Chinese Academy of Sciences, Shanghai, China) were used to evaluate the cell-gel constructs. Cells were expanded in the α-MEM medium supplemented with 10% fetal bovine serum, and 1% penicillin/streptomycin in a 5% CO_2_ humidified atmosphere at 37 °C. The expanded cells were resuspended in PBS for further use.

The selected composite solutions were placed in glass dishes to form a thin layer and the dishes were exposed to UV light at 4 °C for sterilization. For cell seeding, an aliquot of the composite solution was homogeneously mixed with a given volume of cell-containing culture medium to produce a mixture. Different mixtures were produced by altering the cell density and the employed composite solution. The prepared composite solutions and the cell-containing mixtures were used for the follow-up cell experiments.

A Live/Dead assay was performed using MC3T3-E1-containing gels to access the cell viability. The proliferation of MC3T3-E1 cells that were seeded in different gels was accessed with a CCK-8 cell counting kit (Dojindo Molecular Technologies, Gaithersburg, MA, USA). Details for Live/Dead staining and cell proliferation measurements can be found in our previous study [42].

Some cell-gel constructs were cultured for extended durations and the alkaline phosphatase (ALP) activity and type-I collagen synthesis of the seeded MC3T3-E1 cells were measured. At predetermined time intervals, samples were washed with PBS, crushed, and lysed in the lysis buffer at 4 °C. Supernatants were collected and subjected to ALP activity and type-I collagen measurements using an ALP assay kit (Beyotime, Shanghai, China) and a collagen type I ELISA kit (Biological, Salem, MA, USA). The total protein content in cell-gel constructs was detected using a bicinchoninic acid protein assay kit (Beyotime, Shanghai, China).

### 2.8. Statistical Analysis

Data were expressed as mean ± standard deviation. The mean of independent groups was compared using Student’s *t*-test. The difference between groups was tested using one-way ANOVA. Differences were considered to be statistically significant at a *p*-value less than 0.05.

## 3. Results and Discussions

### 3.1. Characterization of CH-CY Conjugates

**Figure 1:**
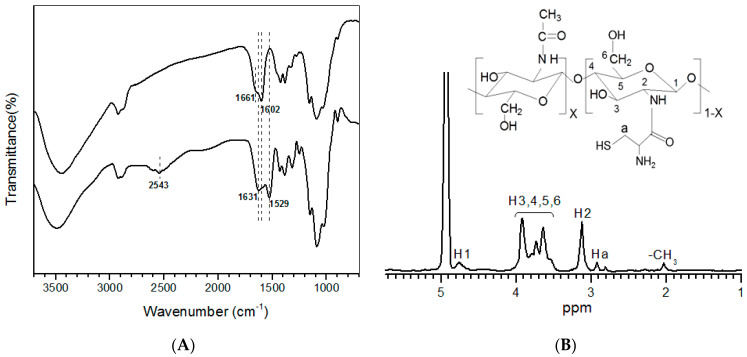
Representative FTIR spectra (**A**) for CH and CH-CY and ^1^H NMR spectrum (**B**) for CH-CY.

Representative FTIR spectra for CH and CH-CY are provided in Figure 1A. The spectrum of CH exhibits absorbance at around 1661 cm^−1^ as a shoulder for carbonyl (C=O) stretching of amide I, indicative of the high DDA feature of CH [39], and a clear absorbance peak at about 1602 cm^−1^ belongs to amide II vibration of CH. In the spectrum of CH-CY, the lower field shift of the peak from 1661 to 1631 cm^−1^ indicates the amide bond formation due to the reaction between the amino group of CH and carboxylic group of CY [19,43]; and a weak absorbance peak registering at 2543 cm^−1^ can be ascribed to S-H stretching [43,44]. Figure 1B shows the ^1^H NMR spectrum for CH-CY. It can be seen that, besides several peaks belonging to CH at *δ*(ppm): 2.04, 3.13–4.91 [22,39,45], a new peak appeared at *δ* (ppm): 2.92, which can be assigned to methylene protons on -CH_2_SH in CH-CY [20,22]. The results in Figure 1 demonstrate that CH-CY conjugate has been successfully synthesized.

Four kinds of CH-CY conjugates were synthesized by changing the molar ratio of the carboxyl group in CY to the amino group in CH while keeping the reaction conditions constant, and several parameters for them are provided in Table 1. The content of thiol groups in CH-CY conjugates was detected to significantly increase as the molar ratio changed from 1.0 to 2.0, and it did not rise with a further increasing molar ratio up to 3.0. CH-CY-c was thus selected for the follow-up gel preparation whenever the CH-CY conjugate was involved.

### 3.2. Parameters for Bioglass Nanoparticles

**Figure 2:**
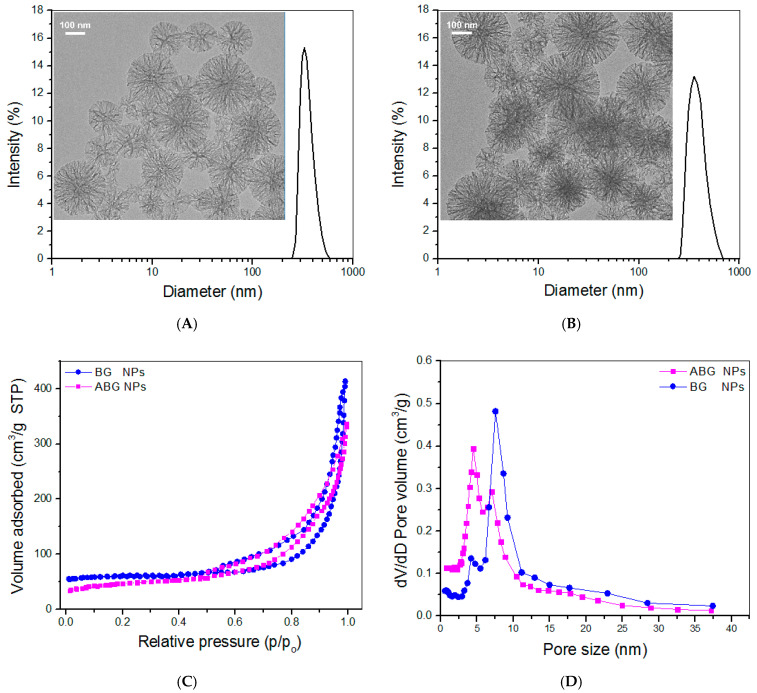
Size distributions (DLS measurement) and TEM micrographs for BG NPs (**A**) and ABG NPs (**B**); and nitrogen adsorption-desorption isotherms (**C**) as well as pore-size distributions (**D**) for BG and ABG NPs.

BG NPs with a high pore volume were first synthesized so that they could be suitable for subsequent drug loading after amino-functionalization. The inserted TEM micrograph in Figure 2A displays that BG NPs were spherical and highly porous with radially porous morphology and good dispersion. Their hydrodynamic size exhibited a nearly symmetrical distribution. After amino-functionalization, the achieved empty ABG NPs were still spherical and well dispersive, and importantly, their radial pore architecture was still clearly visible (Figure 2B), connoting their suitability for subsequent drug loading.

The recorded N_2_ adsorption-desorption isotherms for BG and ABG NPs are presented in Figure 2C. Three characteristics can be drawn from Figure 2C: (i) there were typical hysteresis loops in these isotherms; (ii) the inception turning points in these isotherms were around 0.5 (p/p_0_) and (iii) the isotherm corresponding to BG NPs showed a steeper upward trend in the higher relative pressure range compared to ABG NPs. These characteristics make the point that there are mesoporous pores inside these BG and ABG NPs, and additionally, BG NPs may contain more pores with larger pore sizes in comparison to ABG NPs [46,47]. Curves in Figure 2D exhibit pores in BG and ABG NPs had wide pore-size distributions and some pores were measured to be larger than 10 nm. Several sets of samples for both BG and ABG NPs were measured to determine their parameters and the results are summarized in Table 2.

It can be seen that ABG NPs had a significantly smaller surface area, pore volume, and pore size but had larger hydrodynamic particle size and positive ζ-potential when compared to BG NPs. Because of the highly porous features of BG NPs (Figure 2), DF-P molecules would thus react with the hydroxyl groups on the surface of pores inside BG NPs, and also, with the hydroxyl groups on the surface of BG NPs [47,48], which would result in the reduced surface area, pore-volume, and pore-size for the ABG NPs. BG NPs are known to have negative ζ-potential because of their hydroxyl group-exposed surface. After amino-functionalization, many free amino groups will appear on the surface of the resulting ABG NPs. Consequently, ABG NPs attain positive ζ-potential and relatively enlarged size compared to BG NPs.

Que was loaded into ABG NPs by mainly changing two variables: the concentration of feed Que solutions and the mass ratio of Que to ABG NPs, while maintaining processing conditions unchanged. Several parameters for four kinds of Que-loaded ABG NPs are itemized in Table 3. Data in Table 3 reveal that these Que-loaded NPs had slightly larger mean sizes (*p* > 0.05) but remarkably reduced ζ-potential (*p* < 0.001) when compared to their blank counterpart (Table 2). Although any of these Que-loaded NPs can be used for the construction of ABG/CH-CY composite hydrogels, ABG-Q4 NPs have been selected for the subsequent preparation of composite gels on account of their notably higher DL (*p* < 0.01) compared to the other three kinds of NPs. In addition, only one kind of Que-loaded NPs was employed for building composite gels, which would be conducive to figuring out the effect of the gel matrix on the release patterns of Que and ions.

### 3.3. CH-CY Hydrogels

Previous studies indicated that CH-CY is gelable through thiol-involved linkages without the aid of additional chemical crosslinkers [19,22], and β-glycerophosphate sodium can help to expedite the gelation of CH-CY solution due to the presence of amino groups in the C-2 sites of CH backbone and CY-derived side chains [22]. Nevertheless, so-built CH-CY hydrogels were found to be mechanically weak. Thus, DF-P was used as an additional crosslinker for building amino-bridged networks in the CH-CY gels to enhance their strength. By changing the concentration of CH-CY solutions or the applied amount of DF-P1000, two sets of CH-CY gels were constructed, as illustrated in Table 4. In this study, although CH-CY solutions with a concentration of 3 wt% or even higher were also gellable, such concentrated CH-CY solutions were found to be unsuitable for the subsequent preparation of ABG/CH-CY composite gels because a higher concentrated ABG/CH-CY solution could gelatinize quickly, leading to difficulty in its injection application. The CH-CY solution concentration used for the present gel preparation was thus controlled at 2.5 *w*/*v*% or lower.

**Figure 3:**
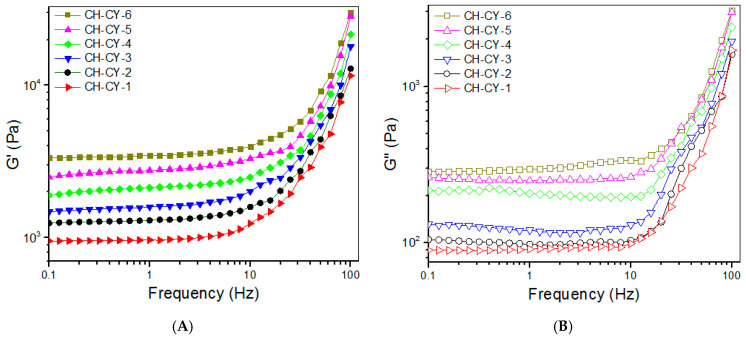

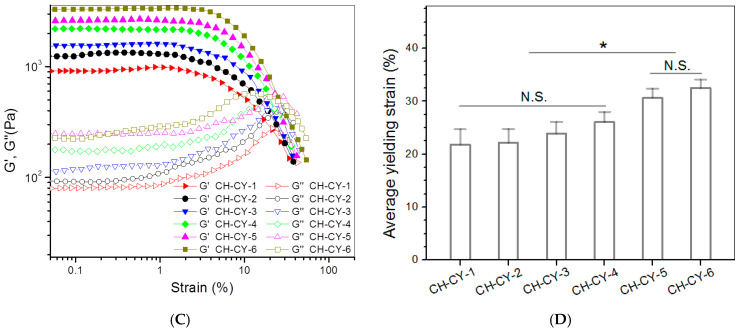
Frequency-dependent changes of G′ (**A**) and G′′ (**B**), strain sweep spectra (**C**), and average yielding strains (**D**) for CH-CY gels (*, *p* < 0.05; N.S., not significant).

Frequency sweep spectra of G′ and G″ for CH-CY gels were detected and the gel strength was compared to each other within the linear viscoelastic region (LVR) with an upper-frequency limit of less than 10 Hz [49]. The curves in Figure 3A exhibit that the G′ value of CH-CY-1, CH-CY-2, and CH-CY-3 gels markedly increased with the increasing concentration of CH-CY solutions, and on the other hand, at a fixed 2.5% CH-CY solution concentration, the G′ value of CH-CY-4, CH-CY-5, and CH-CY-6 gels rose from around 2.0 to 3.3 kPa when the applied amount of DF-P1000 started its linear ascent from 0.4 to 0.6 *w*/*v*%. In Figure 3B, G″ for these gels showed a growing trend similar to their G′, but the difference in G″ was less significant in each set of gels when compared to their G′. To quantitatively compare these gels, many groups of gel samples were measured to determine their average G′ and G″ at 1 Hz, and the obtained data are also provided in Table 4. The magnitude of G′ and G′/G″ ratio can be in principle used for assessing the strength of the hydrogel. In general, a mechanically strong hydrogel has a large G′, and conjointly, its G′ is one order or even two orders of magnitude greater than their G″ [49,50]. CH-CY-1, CH-CY-2, and CH-CY-3 gels have mechanically weak characteristics in view of their low G′ value. CH-CY-4, CH-CY-5, and CH-CY-6 gels show significantly improved strength, but they cannot be considered mechanically strong ones because their G′ value is not high, and the corresponding G′/G″ ratio is about 10 or slightly higher.

Strain sweeps of G′ and G″ for these gels were also detected for evaluating their elasticity using the yielding strain as an indicator [49,50], and strain sweep spectra together with average yielding strains for these gels are represented in Figure 3C,D. CH-CY-1, CH-CY-2, CH-CY-3, and CH-CY-4 gels showed their yielding strains of around 20% or slightly higher without significant differences, indicating their poor elasticity. CH-CY-5 and CH-CY-6 gels had higher yielding strains than other gels, revealing that they have attained some improvements in their elasticity. Despite the establishment of the dual networks inside these CH-CY gels, their strength and elasticity still need to be substantially improved to meet mechanical requirements in bone repair [1,7,9,14]. Based on the results elucidated in Figure 3 and Table 4, Que-loaded ABG NPs were thus incorporated into CH-CY-6 gel for constructing multi-crosslinked composite hydrogels.

### 3.4. Multi-Crosslinked ABG/CH-CY Hydrogels

**Figure 4:**
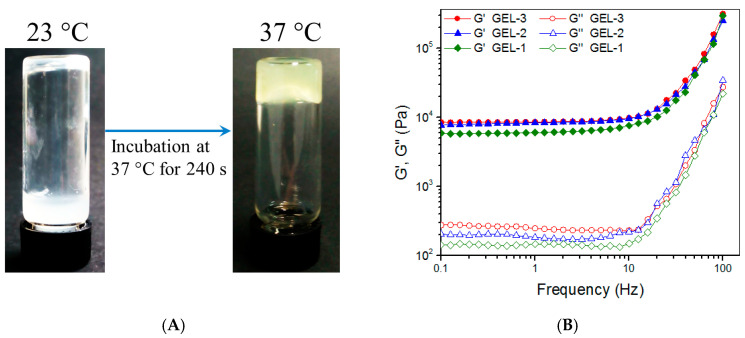

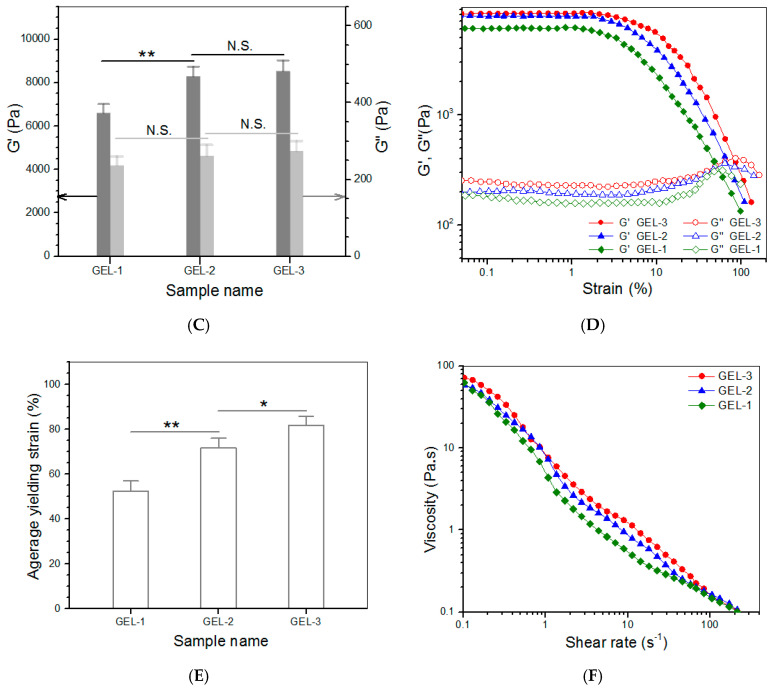
Photos of the sol-gel transition of the composite solution with the same composition as GEL−2 gel (**A**), frequency-dependent functions of G′ and G″ (**B**), average values of G′ and G″ at 1 Hz (**C**), variations in G′ and G″ versus strain (**D**), average yielding strain (**E**) and shear rate dependency of viscosity (25 °C, (**F**)) for gels illustrated in Table 5 (*, *p* < 0.05; **, *p* < 0.01; N.S., not significant).

It is reported that DF-P can crosslink CH via the Schiff base reaction between the terminal benzaldehyde groups in DF-P and amino groups in CH, and the resulting aromatic Schiff bases are much more stable than aliphatic Schiff bases [51]. In our previous study, amino-functionalized mesoporous silica NPs have been effectively cross-linked using genipin, a kind of small molecule crosslinker [52], indicative of the reactiveness of amino groups on the surface of NPs. Hence, the presently employed DF-P would be able to crosslink the amino groups in CH-CY or ABG NPs and contribute to building stable multi-networks in the ABG/CH-CY composite gels. DF-P with various PEG lengths was used in this study, intending to achieve the desired gels with high enough strength and elasticity. To ensure the adequate safety of composite gels, the DF-P dosage was controlled to a level of 0.6 *w*/*v* % or lower. Four kinds of ABG/CH-CY composite gels were thus produced, and their compositional proportions and relevant results are provided in Table 5 and Figure 4, respectively.

Figure 4A shows photos for sol-gel transition of an ABG/CH-CY composite solution. The photos reveal that this solution was flowable at room temperature and able to transform into a gel at 37 °C for around 4 min. The frequency sweep spectra in Figure 4B display that the G′ value in LVR for GEL-1, GEL-2, and GEL-3 gels was much greater than that for their counterpart, CH-CY-6 gel (Figure 3A). Bar-graphs in Figure 4C show that these gels had their G′ of around 6.6, 8.2, and 8.4 kPa, and the G′ of GEL-2 and GEL-3 gels was seen to be significantly higher than that of GEL-1 gel. Importantly, the G′/G″ ratio for GEL-1, GEL-2, and GEL-3 gels reached about 27.9, 31.7, and 31.2, respectively, which were considerably larger than that for CH-CY-6 gel, demonstrating that GEL-1, GEL-2, and GEL-3 gels behave similar to mechanically strong gels because of their high G′ and large G′/G″ ratios. The functions of G′ and G″ versus strain as well as the average yielding strains for GEL-1, GEL-2, and GEL-3 gels are explicated in Figure 4D,E. The bar graphs show that the yielding strain of GEL-1 was higher than 50%, whereas GEL-2 and GEL-3 gels had their yielding strains higher than 70% with a significant difference when compared to GEL-1. In comparison to CH-CY-6 gel (Figure 3D), it can be drawn that GEL-1, GEL-2, and GEL-3 gels have been remarkably improved in their elasticity.

These results are rational because multi-crosslinked networks are constructed inside these ABG/CH-CY composite gels due to the combination of ABG NPs. The benzaldehyde groups in DF-P can react with amino groups in the CH backbone and CY-involved side chains to build a CH-CY alone associated network, and meanwhile, they can also crosslink amino groups that respectively belong to CH-CY and ABG NPs to build another network. Together with the thiol-bridged network, there are at least three networks in ABG/CH-CY composite gels. DF-P molecules have a linear structure and their PEG segment would thus exist inside ABG/CH-CY composite gels in the form of random curls, and these PEG curls will unbend during the gel strain. Given that the DF-P molecules with the long PEG segment are employed for producing ABG/CH-CY composite gels, these DF-P molecules would have more opportunities to randomly entangle with CH-CY molecular chains and interpenetrate into networks when compared to the DF-P molecules having short PEG segment, which could afford ABG/CH-CY composite gels improved strength and enlarged extensibility. Taken together, the establishment of multi-crosslinked networks and the employment of the DF-P with larger Mn of PEG segment (DF-P3400 and DF-P5000) significantly enhance ABG/CH-CY composite gels in their strength and elasticity in comparison to the CH-CY gels.

To examine the injective applicability of GEL-1, GEL-2, and GEL-3 gels, their respective solutions were tested to determine their viscosity versus shear rate, and the results are elucidated in Figure 4F. These composite solutions were viscous in the low shear rate range and showed a similar declining trend in viscosity as the shear rate increased. Their viscosity became markedly low once the applied shear rate reached 10 s^−1^ or higher, indicating that they have shear-thinning features. Considering that the injection of composite solutions is usually conducted at ambient temperature, the results in Figure 4E account for their well-defined injectability.

### 3.5. Release Profiles of Ions and Que

**Figure 5:**
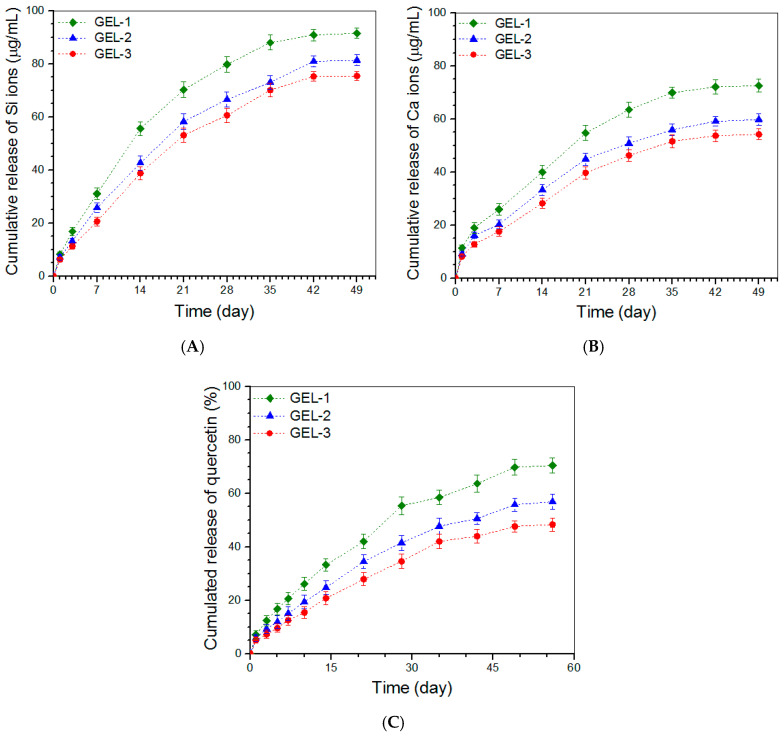
Release profiles of Si ions (**A**), Ca ions (**B**), and quercetin (**C**) for gels illustrated in Table 5.

Ion release patterns for the gels formulated in Table 5 were detected and obtained data are plotted in Figure 5. It is observed from Figure 5A that a Si-ion amount of around 9 μg/mL or less was released from these gels on the first day, and after that, GEL-2 and GEL-3 gels underwent sustained Si ion release at similar release rates in approximately linear manners for around three weeks. Curves in Figure 5B delineate certain burst release features of Ca ions from these gels but show approximately linear release trends starting from around day 3. In both Figure 5A,B, it can be noticed that the difference in release rate between GEL-1 and GEL-2 or GEL-3 became very significant after around 7-day release, whereas GEL-2 and GEL-3 had similar release rates without significant differences. Since these gels contain the same amount of ABG NPs but differ from each other in strength and elasticity (Figure 4), the significantly slower release rates of Si and Ca ions observed from GEL-2 and GEL-3 gels can be ascribed to their higher strength and elasticity compared to that for GEL-1 gel.

Que is practically insoluble in water (<10 μg/mL in water at 25 °C) [53], and generally, has low bioavailability and poor stability. When a hydrogel is employed as the carrier for the local administration of Que, the key issue concerned is how to efficiently load enough amount of Que into the hydrogel and manage its controlled and sustained release. Direct loading of Que into hydrogels with the aid of organic solvents such as ethanol and dimethyl sulfoxide has been explored [33,36,53]. In doing so, the organic solvent should be removed from the resulting hydrogels otherwise such Que-loaded hydrogels may cause potential side effects due to the solvent release; in addition, this kind of Que-loaded hydrogel usually shows a severe initial burst because in many cases, hydrogels constructed with natural polymers have limited ability to administer the controlled and sustained release of drugs or biomolecules [8].

In the present instance, Que was first loaded into ABG NPs and the resulting NPs were then embedded into CH-CY gels. In comparison to some Que-loaded hydrogels reported in the literature [33,36,53,54,55], presently developed Que-loaded composite hydrogels can fulfill several missions at the same time: (I) the Que-load in the composite gels can be tailored by altering either the Que-load in ABG NPs or the content of Que-loaded ABG NPs in the composite gels; (II) ABG NPs would contribute significantly to the mechanical enhancement of the composite gels; and (III) ABG NPs would cooperate with the gel matrix to synergistically modulate the release of Que. Figure 5C presents the Que release profiles for GEL-1, GEL-2, and GEL-3 gels. The profiles exhibit that a Que load of around 7% or less was released from these gels on the first day, and afterward, the release behavior of Que followed different patterns for a few weeks. The GEL-1 gel released the Que in a non-linear upward trend and the cumulative release of Que at the end of 7 weeks reached slightly about 70%. With regard to GEL-2 and GEL-3 gels, Que was seen to release in an approximately linear way for a few weeks starting from day 3. These results indicate that the strong gels have a better capability to slow down the release of Que when compared to that having relatively weak strength.

In this study, by embedding Que-loaded ABG NPs into CH-CY gel, the resulting composite gels show an ability to administer the Que release in a regulatory manner with sustained release of quercetin for a period of around 7 weeks. In GEL-1, GEL-2, and GEL-3 gels, the Que-loaded ABG NPs with surface-exposed amino groups would be cross-linked with CH-CY to some extent by the DF-P crosslinker that corresponds to each of the three gels, or physically entangled with CH-CY chains, which will lead to the formation of a capping layer wrapped on the surface of Que-loaded ABG NPs. As a result, Que molecules have to diffuse out of the capping layer of ABG NPs first and then pass through the gel matrix to reach the release medium. During the passage of Que molecules through the gel matrix, the gels having higher strength may exert higher resistance to Que molecules due to the existence of higher crosslinking or more chain entanglement in these gels, thus leading to slower Que release. It is worth mentioning that many preliminary experiments had been conducted in this study to optimize parameters respectively matching with ABG NPs and ABG/CH-CY gels, which finally results in approximately linear Que release.

To date, numerous release systems have been developed for delivering drugs, proteins, peptides, therapeutic ions, and genes. Despite the differences in composition, structure, and function of these delivery systems, several empirical models have been established to describe their release kinetics [56,57,58]. In the case of swellable polymer matrices, the kinetic behavior of drug release can be estimated from the following semi-empirical equation [56,59]:

*M*_t_/*M*_∞_ = *kt*^n^ or log[*M*_t_/*M*_∞_] = log*k* + *n*log*t* (2)

where *M*_t_/*M*_∞_ is the fractional release of the drug at time *t*; *k* is a constant correlated to the structural and geometric characteristics of the release system as well as the release rate; and *n* is the release exponent, indicative of the drug release mechanism. Based on Equation (2), *n* and *k* for different gels were calculated and relevant data are summarized in Table 6.

Generally speaking, the *n* value less than 0.45 is indicative of a Fickian diffusion mechanism; the *n* value ranging between 0.45 and 0.85 indicates anomalous transport controlled by both diffusion and swelling; the *n* value larger than 0.85 signifies super case-II transport which is related to polymer relaxation during swelling [56,57,58]. Data in Table 6 show that all tested gels had their *n* values changing between 0.51 and 0.7, revealing that the release of Si ions, Ca ions and Que from these gels follows an anomalous transport mechanism modulated by both Fickian diffusion and swelling.

### 3.6. Cell Growth and Analysis

**Figure 6:**
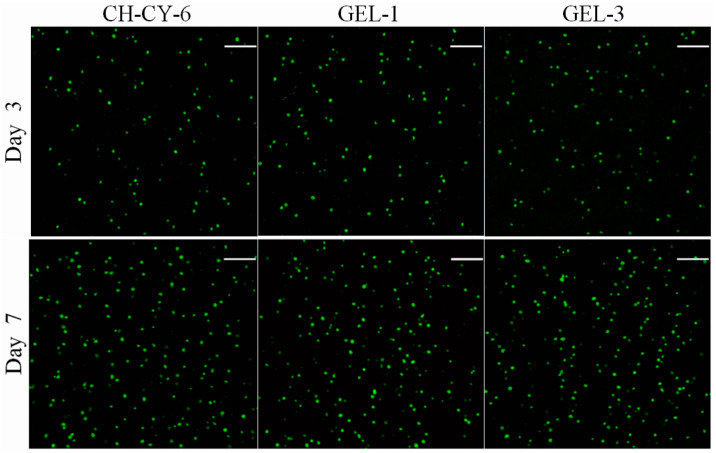
Confocal micrographs for stained MC3T3-E1 cells that were seeded in different gels (green: viable cells; red: dead cells; scale bar: 150 μm).

The CH-CY-6 gel differs from the GEL-1 gel in composition, and in addition, the crosslinkers used in GEL-1 and GEL-3 gels are different in the length of PEG segments. Thus, CH-CY-6, GEL-1, and GEL-3 gels were selected for the in vitro evaluation. Figure 6 presents representative fluorescence micrographs for the stained MC3T3-E1 cells that were seeded in different gels and cultured for various periods of up to 7 days. Very few dead cells were detected from these gels after the cell-gel constructs were cultured for 3 or 7 days, respectively. Moreover, the cell density in the micrographs corresponding to the 7-day culture was remarkably higher than that assigned to the 3-day culture. These micrographs signify that the seeded MC3T3-E1 cells had high viability, and also, the gels showed similar abilities to support the growth of seeded cells despite their differences in composition or cross-linker applied.

**Figure 7:**
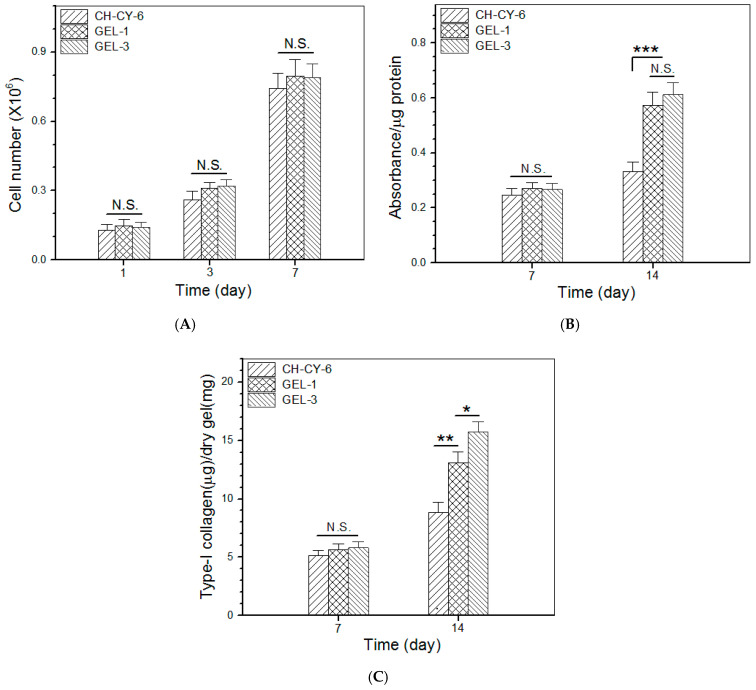
Proliferation (A), ALP activity (**B**) and type-I collagen synthesis (**C**) of MC3T3-E1 cells growing inside gels (*, *p* < 0.05; **, *p* < 0.01; *** *p* < 0.001; N.S., not significant).

Figure 7A elucidates results for cell proliferation in different gel matrices. The bar graphs delineate that the cell growth experienced two distinct phases: slow cell growth from day 1 to day 3; and pronouncedly fast cell growth from day 3 to day 7. The first phase can be attributed to cell attachment with population recovery, and the second phase confirms the occurrence of cell proliferation. Figure 7A also exhibits that there were no significant differences in the cell number among these gels, suggesting that they have similar capabilities to support cell proliferation.

ALP activity is known to be a common indicator for evaluating the early stage osteogenic development of osteoblast-like cells [1,60]. The cell-gel constructs cultures for various durations were submitted for ALP activity detection by an ALP essay, and the obtained data are depicted in Figure 7B. It can be observed that there was no significant difference in the ALP activity for the cells seeded in these gels after 7-day culture. After extended culture to 14 days, the ALP activity for GEL-1 and GEL-3 gels was shown to be similar without significant differences, but it was remarkably higher than that belonging to the cells seeded in CH-CY-6 gel. As shown in Table 4 and Table 5, the key difference between the CH-CY-6 gel and the other two composite gels is that the CH-CY-6 did not contain any Que-loaded ABG NPs. The ionic dissolution products of BG NPs composed of CaO and SiO_2_, usually including Si and Ca ions, are known to have osteogenic activity [23,28], and the Que is also demonstrated to show the capability to promote osteogenesis [32,35,38]. Accordingly, the considerably higher ALP activity detected from GEL-1 and GEL-3 gels should be attributed to the combined contribution of released ions and Que.

Type-I collagen synthesized by the osteoblast-like cells is often used as another indication for assessing the osteogenic development of the cells [1,61]. The amount of type-I collagen in the cell-seeded gels was measured, and relevant data are plotted in Figure 7C. The bar graphs display that the deposited type-I collagen amount was similar among these gels after 7-day culture. During this period of culture, the seeded cells need to go through the process of adhesion, restorative growth, and subsequent proliferation. A 7-day growth period might be too short for them to synthesize a large amount of type-I collagen, thus causing an insignificant difference in their type-I collagen deposition. As the culture time advanced from day 7 to day 14, the amount of deposited type-I collagen was seen to rise at varied rates among these gels. The deposited amount of type-I collagen in GEL-1 gel was measured to be much higher compared to CH-CY-6 gel on day 14. GEL-1 gel differed from CH-CY-6 gel in the ingredient of Que-loaded ABG NPs as well as in strength and elasticity. Thus, it can be inferred that Que-loaded ABG NPs and much higher strength and elasticity in GEL-1 gel synergistically contribute to its high type-I collagen deposition compared to CH-CY-6 gel. With respect to the difference in the synthesized type-I collagen amount between GEL-1 and GEL-3 gels, the significantly higher type-I collagen deposition in GEL-3 gel should be attributed to the higher strength and elasticity of GEL-3 gel when compared to the GEL-1 gel. The present study was focused on investigations into the physicochemical properties, controlled ion and drug release, biocompatibility, and osteogenic potency of these newly developed composite gels. Further studies related to their application in bone repair are in progress. The relevant results will be presented in separate reports.

## 4. Conclusions

A new type of multi-crosslinked network composite hydrogel with certain strong and elastic characteristics was successfully constructed using CH-CY conjugate and Que-loaded ABG NPs while employing DF-P with varying PEG segment lengths as a crosslinker. The formulated ABG/CH-CY composite hydrogels showed much higher strength and elasticity in comparison to the gels composed of a single CH-CY component. PEG segment length in DF-P was found to impose significant impacts on the properties of resulting hydrogels, and the gel cross-linked by a DF-P crosslinker with a long PEG segment would endow the resulting ABG/CH-CY gel with significantly higher strength or elasticity when compared to those cross-linked by a DF-P crosslinker having a short PEG segment. Moreover, some ABG/CH-CY gels with higher strengths and elasticities exhibited the ability to release Si or Ca ions and the loaded Que in approximately linear manners for a few weeks. ABG/CH-CY gels were capable of supporting the growth and matrix synthesis of the seeded osteoblast-like cells. The higher strength and elasticity of the gels were conducive to the synthesis of type-I collagen. The results demonstrate that these newly developed ABG/CH-CY hydrogels have the potential for applications in bone repair and regeneration.

## Figures and Tables

**Table 1:** Parameters for CH-CY conjugates.

Sample Name	Molar Ratio of -COOH in CY to -NH_2_ in CH	Content of Thiol Groups (µmol/g) ^(a)^	Substitution Degree of Thiol Groups (%) ^(b)^	Solubility in Water ^(c)^
CH-CY-a	1	56.8 ± 4.31	2.44	+
CH-CY-b	1.5	103.4 ± 7.52	4.45	+
CH-CY-c	2	149.2 ± 10.14	6.32	+
CH-CY-d	3	146.3 ± 11.69	6.27	+

^(a)^ Content of thiol groups in CH-CY was determined by Ellman’s method. ^(b)^ The substitution degree of thiol groups in CH-CY was determined by ^1^H NMR. ^(c)^ “+” indicates the full solubility of CH-CY conjugates (2.5 wt%) in water.

**Table 2:** Parameters for different kinds of nanoparticles.

Sample Name	Surface Area (m^2^/g)	Pore Volume (mL/g)	Pore Size (nm)	ζ-Potential (mV)	Particle Size (nm) ^(b)^	Content of Amino Groups (μmol/mg)
BG ^(a)^	579.4 ± 43.6	1.53 ± 0.11	9.47 ± 0.69	−13.6 ± 1.02	341.6 ± 13.8	−
ABG	416.1 ± 39.2	1.08 ± 0.09	7.53 ± 0.51	30.7 ± 1.38	367.2 ± 23.6	0.573 ± 0.042

^(a)^ CaO/SiO_2_ molar ratio for BG NPs was detected to be around 0.13 from their energy dispersive spectra ^(b)^ Data were obtained via DLS measurements.

**Table 3:** Parameters for quercetin-loaded ABG nanoparticles.

Sample Name	Ratio of Que to ABG NPs (wt./wt.)	Que Concentration (mg/mL)	DL (%)	Particle Size (nm) ^(a)^	ζ-Potential (mV)
ABG-Q1	1.0	100	10.37 ± 0.52	382.6 ± 22.54	10.4 ± 0.31
ABG-Q2	1.0	200	13.29 ± 0.47	391.1 ± 23.47	9.8 ± 0.43
ABG-Q3	2.0	100	14.84 ± 0.61	404.7 ± 20.16	9.2 ± 0.52
ABG-Q4	2.0	200	18.26 ± 0.73	409.3 ± 28.63	8.7 ± 0.49

^(a)^ Data were obtained via DLS measurements.

**Table 4:** Parameters for hydrogels without incorporating nanoparticles.

Sample Name	CH-CY (*w*/*v* %)	DF-P1000 (*w*/*v* %)	G′ at 1 Hz (Pa)	G″ at 1 Hz (Pa)
CH-CY-1	1.5	0.3	962.5 ± 51.4	81.4 ± 6.1
CH-CY-2	2.0	0.3	1283.2 ± 82.6	93.5 ± 7.3
CH-CY-3	2.5	0.3	1561.8 ± 94.8	116.2 ± 8.9
CH-CY-4	2.5	0.4	2079.3 ± 129.1	217.8 ± 16.2
CH-CY-5	2.5	0.5	2647.1 ± 136.2	253.7 ± 20.6
CH-CY-6	2.5	0.6	3312.4 ± 172.3	265.3 ± 22.4

**Table 5:** Composition proportions for composite hydrogels.

Sample Name	CH-YC (*w*/*v* %)	ABG-Q4 NPs (*w*/*v* %)	DF-P1000 (*w*/*v* %)	DF-P3400 (*w*/*v* %)	DF-P5000 (*w*/*v* %)
GEL-1	2.5	2.0	0.6	−	−
GEL-2	2.5	2.0	−	0.6	−
GEL-3	2.5	2.0	−	−	0.6

**Table 6:** Kinetic parameters for hydrogels.

Sample ^(a)^	*k*	*n*	*r* ^2^
GEL-1 (Si ion)	8.39	0.67	0.9926
GEL-2 (Si ion)	6.91	0.67	0.9959
GEL-3 (Si ion)	5.64	0.7	0.9935
GEL-1 (Ca ion)	10.78	0.51	0.9913
GEL-2 (Ca ion)	8.81	0.51	0.9884
GEL-3 (Ca ion)	7.45	0.52	0.9849
GEL-1 (Que)	6.59	0.61	0.9961
GEL-2 (Que)	5.01	0.61	0.9887
GEL-3 (Que)	4.07	0.62	0.9819

^(a)^ See Table 5 for the definition of sample names.

## Data Availability

The data presented are available on request from the corresponding author.
